# Life-Threatening Rupture of an Idiopathic Left Hepatic Artery Pseudoaneurysm Successfully Treated with Endovascular Coil Embolization

**DOI:** 10.1155/2020/8835573

**Published:** 2020-09-09

**Authors:** Jorge E. Sandelis-Pérez, Andrés Córdova-Toro, Steven García-Santiago, Erica G. Otero-Cárdenas, Pedro Gil de Rubio-Cruz, Suheiry Márquez-Márquez, Mary J. Rodríguez-Malavé, Arelis Febles-Negrón, José A. Colon-Márquez, Alejandro Hidalgo-Ríos

**Affiliations:** ^1^Internal Medicine Department, University of Puerto Rico-Medical Sciences Campus, San Juan, Puerto Rico; ^2^Radiology Department, University of Puerto Rico-Medical Sciences Campus, San Juan, Puerto Rico

## Abstract

Hepatic artery pseudoaneurysm is a rare condition; they are multifactorial, most of them locating in the extrahepatic vasculature and the mortality associated to its rupture may reach up to 70%. We report a 77 years old female who was admitted due to headache and uncontrolled hypertension and that on her second hospital day developed sudden hemodynamic instability, abdominal pain, fatigue, skin-mucosa pallor, and anemia. Abdominal CT scan with contrast showed a left hepatic artery pseudoaneurysm associated with extensive hemoperitoneum. Patient required emergent hemodynamic stabilization and finally was treated successfully with a superselective endovascular coil embolization. Our patient represents an atypical case of a spontaneous rupture of an idiopathic hepatic artery pseudoaneurysm. Hence, the importance of having a high index of clinical suspicion. Endovascular coil embolization has become the first-line treatment.

## 1. Introduction

Hepatic artery pseudoaneurysm (HAP) is an unusual and potentially fatal condition; as most of them are asymptomatic, the real incidence is unknown [1]. They are multifactorial, but iatrogenic injuries and abdominal trauma constitute the main predisposing factors. Its risk of rupture is higher compared with true aneurysm [2]. The first case was described by James Wilson in 1809 during an autopsy after its rupture [3]. The mortality rate reported after HAP rupture may reach up to 70% [4]. Here, we report the rupture of an idiopathic pseudoaneurysm of left hepatic artery, treated successfully with a superselective endovascular coil embolization.

## 2. Case Report

**Figure 1:**
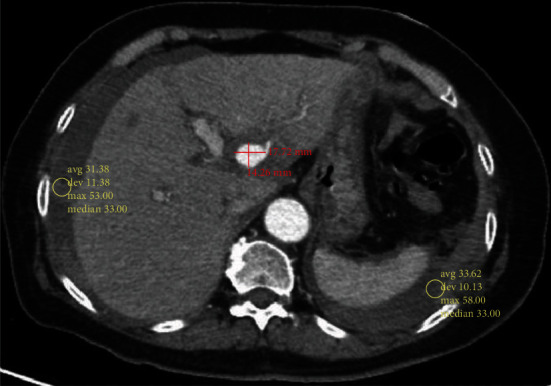
Axial view of CT abdomen and pelvis with IV contrast. There is a pseudoaneurysm from the left hepatic artery going to the segment II, measuring approximately 1.4 cm × 1.8 cm × 1.9 cm. Also, pertinent for an extensive hemoperitoneum particularly around the liver, around the spleen, through right and left paracolic gutter, and in the pelvis.

77-year-old female patient with a past medical history of hypertension and hyperlipidemia came to the hospital complaining of headache since 7 days ago; unilateral, right side, pressure-like, 10/10 in intensity, worsening with movements without relieving factors, headache was accompanied by palpitations, nausea, and one nonbloody vomiting episode; blood pressure had been high during those episodes, last time B/P: 200/100 mmHg, requiring optimization of her antihypertensive treatment. The patient was admitted to complete headache work up, blood pressure control, and symptomatic treatment. Head computed tomography (CT) angiogram was pertinent for bilateral intracranial internal carotid arteries atherosclerotic disease, causing no significant/flow-limiting stenosis. On the second hospital day, the patient started with abdominal pain over the right upper quadrant and left lower quadrant, accompanied by vomiting, shortness of breath, and worsening fatigue. On physical exam, the blood pressure: 85/60 mmhg, heart rate: 100, respiratory rate: 17, temperature: 37, and saturating at 99% (room air), the patient found diaphoretic with dry oral mucosa, conjunctival and mucosa pallor, diffuse abdominal tenderness, and without rebound tenderness or guarding, bowel sound was present. The laboratory tests were pertinent for a sudden drop in hemoglobin levels (almost 7 grams in less than 24 hours), accompanied by leukocytosis, metabolic acidosis with intact renal function, and mild troponinemia. Emergent abdominal CT scan with contrast showed a left hepatic artery pseudoaneurysm associated with extensive hemoperitoneum (Figure 1), for which general surgery was consulted recommending an interventional radiology (IR) evaluation.

**Figure 2:**
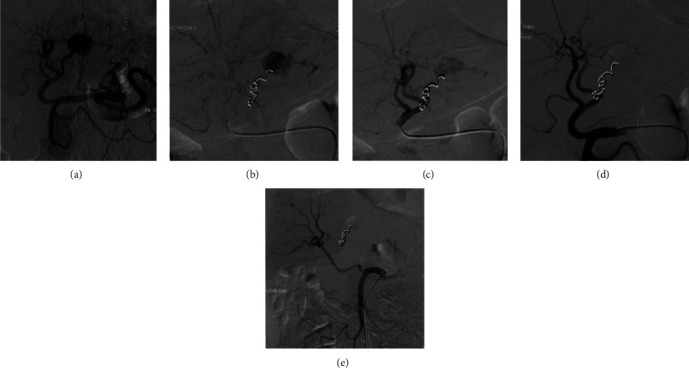
Hepatic and mesenteric arteriogram. Large pseudoaneurysm seen at the left hepatic artery to segment 2/3 of the liver (a). Postcoil embolization of the superselective branch of the left hepatic artery to segments 2/3 of the liver (b). Postembolization control run to the treated vessel showing decreased blood into the pseudoaneurysm and preserved blood flow into the segments 4a/4b of the liver (c). Final control run postcoil embolization showing no further filling of the pseudoaneurysm to segment 2/3 and preserved flow into the remaining left hepatic artery (d). Selective SMA run showing replaced right hepatic artery to the SMA with preserved flow into the right liver and no evidence of backdoor filling into the left hepatic artery pseudoaneurysm (e).

Patient improved hemodynamically with fluid therapy, requiring also several packed red blood cells (PRBC) transfusions and acidosis resolved with Buffer therapy; however, the hemoglobin levels continued in decreasing trend despite blood transfusion, so patient was scheduled for an emergent hepatic and mesenteric arteriogram which identified a large aneurysm from one of the smaller tributaries of the left hepatic artery (LHA) measuring 19 × 16 mm to segment 2 of the liver; a super selective catheterization of the LHA tributary to the aneurysm was done with 5 fr hydrophilic glide cobra catheter; a successful embolization of the feeding vessel to the aneurysm was performed with 2 coils 0.035 fibered 3 × 5 mm and gelfoam slurry; a postembolization control run demonstrates no filling of the aneurysm from back door or any other feeders (Figure 2).

## 3. Discussion

A pseudoaneurysm is a collection of blood that forms between the two outer layers of an artery: the tunica media and adventitia, existing a direct communication of blood flow between the vessel lumen and the aneurysm lumen, being the risk of rupture higher compared with true aneurysm [2]. The HAP rupture can occur in up to 76% [5].

Most frequent described etiologic factors for HAP are iatrogenic injury due to pancreatic, biliary, or hepatic procedures, penetrating or blunt abdominal trauma, recent orthotopic liver transplant, liver biopsy, infections, inflammatory pathologies such as acute pancreatitis, and due to atherosclerosis [6]. Our patient did not have any of the predisposing risk factors apart from atherosclerotic disease.

Anatomically, the HAP can be divided into intrahepatic and extrahepatic [7], the extrahepatic represent 80% of all cases, which are most commonly associated to local infection [8, 9], while intrahepatic are mostly associated to liver puncture due to previous invasive procedure [10].

Nowadays, with widespread access to better diagnostic imaging, most cases of HAP are discovered incidentally during a diagnostic study [4]; however, 80% of cases reported previously presented with rupture as first manifestation [11]. Possible symptoms are nausea, right upper quadrant pain radiating to the back; once the pseudoaneurysm ruptures into the biliary tree, we can observe Quincke's classic triad (jaundice, biliary colic, and gastrointestinal bleeding) characteristic of hemobilia, only seen in one-third of patient, Nevertheless, it is described that 20-30% of them rupture into the peritoneal cavity [4, 8, 12] causing an intra-abdominal hemorrhagic emergency, with a mortality rate at this point reaching up to 82% [13, 14].

Early diagnosis is still the most important element to improve results, among the existing imaging modalities we have Color Doppler Ultrasound which can be performed bedside without radiation exposure but has the limitation of low ultrasound window and is not effective in identifying all ruptured visceral aneurysms as well as identifying retroperitoneal bleeding. On the other hand, CT Angiography is much more accurate in identifying and describing the presence of HAP and its complications [7]; however, conventional DSA continues to be the gold diagnostic standard, especially if therapeutic intervention will be performed [15].

Regarding management, it is important to keep in mind that all hepatic pseudoaneurysms at the time of diagnosis, regardless of size and symptoms, must be treated, because the mortality once it breaks is very high [16]; if the patient presents with hemodynamic collapse secondary to massive blood loss in the setting of a spontaneous rupture of the pseudoaneurysm, the first step is aggressive resuscitation.

Among available therapeutic options, we have arterial reconstructions, endovascular treatments, and retransplantation of the hepatic artery [17]. However, in recent years, the endovascular approach with coil embolization has positioned itself as the first-line treatment in this population with excellent outcomes [14, 18]; it is safer and more precise for locating the pseudoaneurysm and evaluating the collateral flow [19]. Surgery is indicated for hemodynamically unstable patients, embolization failure or rebleeding [20].

## 4. Conclusion

Our patient represents an atypical case of hepatic artery pseudoaneurysm, given the absence of most common predisposing factors. Hence, the importance of having a high index of suspicion for any patient with abdominal pain and hemodynamic instability, the timely diagnosis and management can reduce morbidity and mortality. Endovascular coil embolization has become the first-line treatment.

## Data Availability

Data sharing is not applicable to this article; the patient's medical information can be found on hospital EMR but are not publicly available.
